# Supplementary material for: Impact of the COVID-19 Lockdown in a European Regional Monitoring Network (Spain): Are We Free from Pollution Episodes?
Source: Int J Environ Res Public Health. 2021 Oct 20;18(21):11042. doi: 10.3390/ijerph182111042 (PMC8583473; doi:10.3390/ijerph182111042)
Supplement: Supplementary file 1 [file ijerph-18-11042-s001.zip › ijerph-1375781-supplementary.pdf]

International Journal of
Environmental Research and Public Health

MDPI

Impact of the COVID-19 Lockdown in a European Regional Monitoring Network (Spain): Are We Free from Pollution Episodes?

Gotzon Gangoiti *, Maite de Blas, Maria Carmen Gómez, Ana Rodríguez-García, Eduardo Torre-Pascual, Estíbaliz García-Ruiz, Estibaliz Sáez de Cámara, Iñaki Zuazo, José Antonio García and Verónica Valdenebro

Faculty of Engineering, University of the Basque Country UPV/EHU, 48013 Bilbao, Spain;
* Correspondence: g.gangoiti@ehu.eus, Tel.: +34-94-601-4088; Fax: +34-94-601-7800

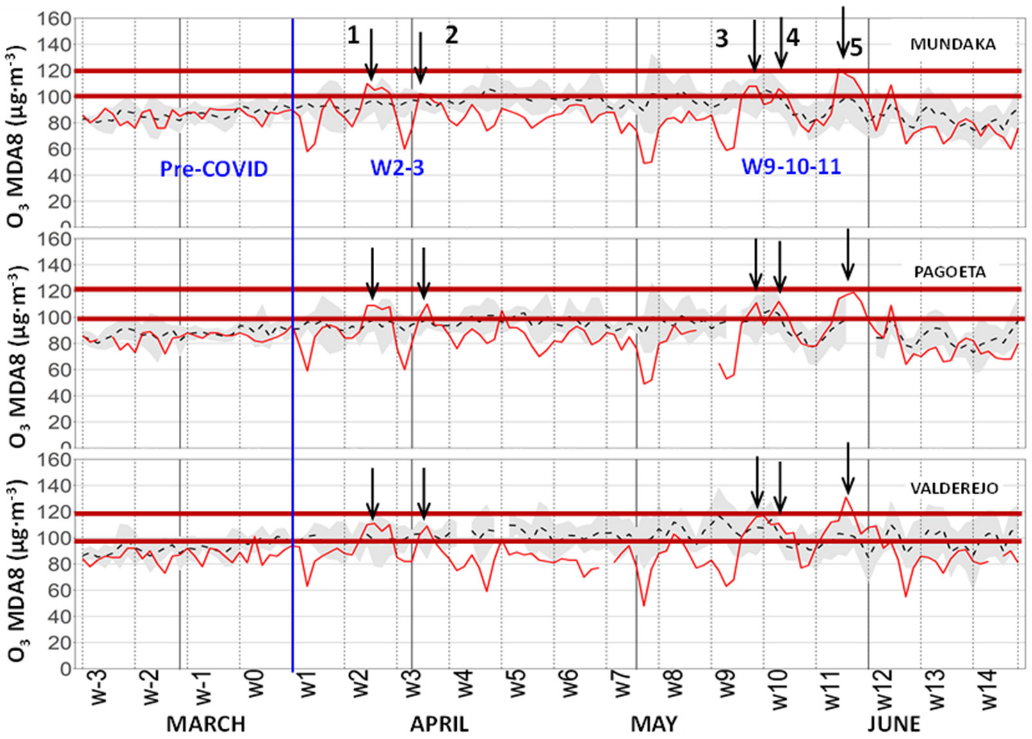

Figure S1: As in Figure 7, MDA8 ozone daily series during a a pre-COVID19 four-week period the lockdown period (solid red line) and during in the three background rural sites, together with the inter-annual averages (dashed lines) and their standard deviations (grey shading). The five simultaneous O_3 exceedances (marked with arrows) of the WHOAQG of 100 $\mu g \cdot m^{-3}$ are also detected in the three rural sites.

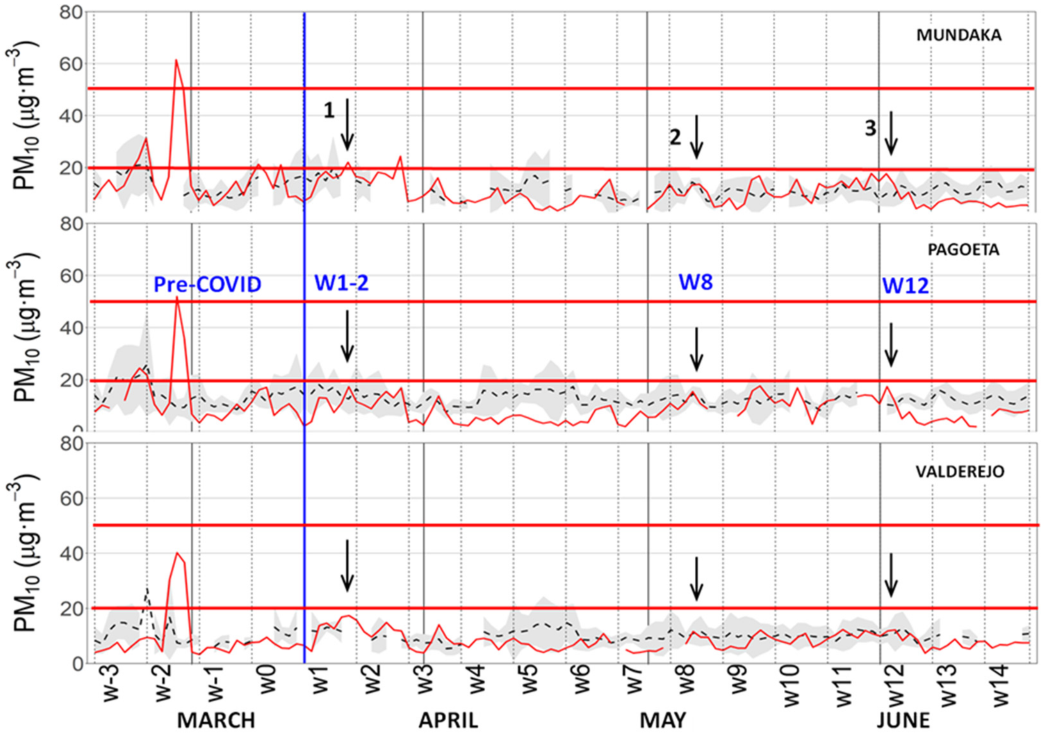

Figure S2. As in Figure 8, 24-hour average concentration series of PM_{10} during pre-COVID four-week period and the lockdown period (solid red line) in the three background rural sites, together with the inter-annual averages (dashed lines) and their standard deviations (grey shading). The PM_{10} episodes observed in the urban sites (marked with arrows) above the WHOAQG reference level of 20 µg·m^{-3} are not detected in the rural sites.

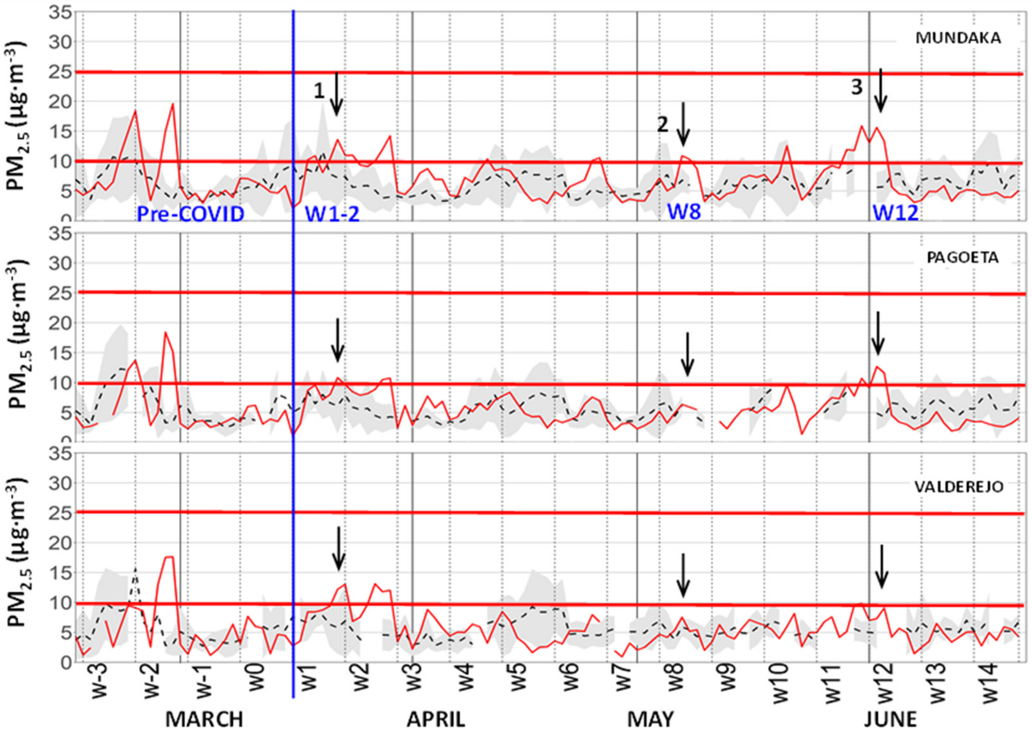

Figure S3. As in Figure 9, 24-hour average concentration series of $PM_{2.5}$ during a pre-COVID four-week period and the lockdown period (solid red line) in the three background rural sites, together with their inter-annual averages (dashed lines) and standard deviations (grey shading). The same three PM_{10} episodes marked in Figure 8 and 9 (arrows) can also be detected in these $PM_{2.5}$ series (above the WHOAQG of 10 $\mu g \cdot m^{-3}$), but not in all three rural sites.
